# PD-1-siRNA Delivered by Attenuated *Salmonella* Enhances the Antitumor Effect of Chloroquine in Colon Cancer

**DOI:** 10.3389/fimmu.2021.707991

**Published:** 2021-07-06

**Authors:** Shuya Lu, Jianhui Gao, Huijie Jia, Yang Li, Yongbin Duan, Fuyang Song, Zhiang Liu, Shuai Ma, Mingyong Wang, Tiesuo Zhao, Jiateng Zhong

**Affiliations:** ^1^ Department of Pathology, The First Affiliated Hospital of Xinxiang Medical University, Xinxiang, China; ^2^ Department of Pathology, Xinxiang Medical University, Xinxiang, China; ^3^ College of Forensic Medicine, Xi’an Jiaotong University Health Science Center, Xi’an, China; ^4^ Institute of Precision Medicine, Xinxiang Medical University, Xinxiang, China; ^5^ Xinxiang Key Laboratory of Tumor Vaccine and Immunotherapy, Xinxiang Medical University, Xinxiang, China; ^6^ Laboratory Medicine, Xinxiang Medical University, Xinxiang, China; ^7^ Laboratory of Molecular Biology of Tumor Reversal, Xinxiang Medical University, Xinxiang, China

**Keywords:** colon cancer, *Salmonella*, chloroquine, PD-1, siRNA

## Abstract

The widespread appearance of drug tolerance and the low efficiency of single treatment have severely affected the survival time of the patients with colorectal cancer. Exploring new treatment options and combined treatment strategies have become the key to improving the prognosis. The combination of immunotherapy and chemotherapy have shown good clinical expectations. Here, we studied the cooperative effects of chloroquine, an anti-malarial drug that is now widely used in anti-tumor research, and RNA interference (RNAi) targeting the immune checkpoint molecule Programmed Death-1 (PD-1) delivered with attenuated *Salmonella*. Our results show that chloroquine can not only significantly inhibit the survival of colon cancer cells and induce apoptosis, but also effectively inhibit cell invasion and migration. The results of *in vivo* experiments show that chloroquine can increase the expression of PD-1 in tumor tissues. Combining chloroquine and PD-1 siRNA can further inhibit the growth and metastases of colon cancer and induce apoptosis. The mechanism underlying this phenomenon is the occurrence of chloroquine-induced apoptosis and the effective immune response caused by the attenuated *Salmonella* carrying PD-1 siRNA. This study suggests that the combined application of PD-1-based immunotherapy and anti-cancer drugs has become a new expectation for clinical treatment of colorectal cancer.

## Introduction

Colorectal cancer (CRC) is the third most common malignant tumor in men and women worldwide, and it is also one of the leading causes of cancer-related death (1, 2). Although traditional treatment, such as surgery, radiotherapy and chemotherapy, can effectively prolong the survival time of patients with colorectal cancer on early stage, it shows poor efficacy for patients with distant metastasis and old age (3). More unfortunately, patients with colorectal cancer have developed significant chemoresistance, both primary and secondary drug resistant (4, 5). To explore a more effective and targeted treatment strategy is the key to improving the survival rate of colorectal cancer patients. Studies have shown that tumor immune escape is the key to the rapid development and metastasis of cancer. Based on this, in recent years, cancer immunotherapy has become a hot spot in cancer treatment research, and it is also a more effective and widely applicable treatment method. Among many immunotherapy methods, immune checkpoint therapy is the most broadly studied. Its mechanism is to improve the anti-tumor immune response by inhibiting the immunosuppressive molecules on the surface of thymus dependent lymphocytes (T cells). Among them, cytotoxic T lymphocyte-associated protein 4 (CTLA-4) or programmed cell death 1 (PD-1) are the most widely studied and applied (6–10).

PD-1, also named CD279, is an immunosuppressive receptor, which regulates the activation of T cells by binding to its ligand Programmed death receptor ligand (PD-L1) (11). Studies have shown that PD-1 is mainly expressed on the surface of immune cells, including CD4^+^, CD8^+^ T and natural killer (NK) cells, while tumor cells is mainly expressed PD-L1 (12). The combination of PD-L1 molecules on the surface of tumor cells and PD-1 molecules on the surface of immune cells inhibits the anti-tumor immune response of immune cells and mediates the occurrence of immune escape of tumor cells (13). Therefore, PD-1 and PD-L1 have become the key target molecules of immunotherapy. Based on this theory, a variety of monoclonal antibodies targeting PD-1 have been developed worldwide, including opdivo (nivolumab) and keytruda (pembrolizumab), which have been used in clinical practice (14, 15). In addition to monoclonal antibodies, small molecule inhibitors and siRNA based on PD-1 have also entered preclinical research (11, 16). We have also reported attenuated *Salmonella* carrying PD-1 siRNA in the treatment of melanoma (17). At present, PD-1/PD-L1 inhibitors are combined with chemotherapy, radiotherapy, targeted drug therapy and other immunotherapies, which increases the number of CD8^+^ T cells in the tumor microenvironment of patients, destroys the immune escape of tumors, and enhances the anti-tumor effect of PD-1/PD-L1 inhibitors (18–20).

Chloroquine (CQ) is a wide range of anti-malarial drugs. In addition to malaria, chloroquine is currently the most common drug used to relieve acute and chronic inflammatory diseases, such as rheumatoid arthritis (21), systemic lupus erythematosus (22, 23), and Knot disease (24). Studies have found that chloroquine also can inhibit cell cycle, autophagy and induce apoptosis in lung cancer, liver cancer, and gallbladder cancer, which has good anti-tumor potential (25–28). In addition, it is reported that chloroquine can be used in combination with other chemotherapy to enhance its anti-tumor effect (29). However, the relationship between chloroquine and immunotherapy for tumors is hitherto unclear.

In this study, we first determined the effect of chloroquine on the survival, proliferation and apoptosis of colorectal cancer cells in *in vitro* experiments. To clarify the regulation of immune checkpoint by chloroquine, we established a subcutaneous transplanted-tumor model of colorectal cancer in mice. The results showed that chloroquine could increase the expression of PD-1 in tumor tissues while inducing tumor growth inhibition. Chloroquine combined with PD-1 siRNA delivered with attenuated *Salmonella* could significantly enhance the tumor growth inhibition through upregulation of the number and activity of immune cells in tumor tissues.

## Materials and Methods

### Cell Lines, Mice and Bacteria

Mouse colon cancer cell line, CT26, was obtained from American Type Culture Collection (ATCC, Rockville, MD). Human colon cancer cell lines, RKO and HCT-116 were obtained from Procell (Wuhan, China). BALB/c mice (6-8 weeks, female rat) were purchased from Charles River Laboratories (Beijing, China). RKO, HCT-116 and CT26 cell lines were cultured in high glucose DMEM with 10% FBS, 1% Penicillin streptomycin (all purchased from Gibco, USA). The experimental mice were fed with food and water regularly, Animal experiments were approved by the ethics committee of Xinxiang Medical University (Xinxiang, China). Recombinant attenuated *Salmonella* (containing siRNA-PD1 or siRNA-Scramble plasmids with phP/phQ deletion) has been constructed and stored in our laboratory. The sequence of siPD-1 is as follows: GATCCGGGTTTGAGCCAACCCGTCCAGTTC AAGAGACTGGACGGGTTGGCTCAAACCTTTTTTGGAAA.

### Cell Proliferation and Viability Assay

The RKO, HCT-116 and CT26 cells in the logarithmic growth phase were plated in a 96-well plate (1.2×10^4^ cells/well for proliferation assay, 0.4×10^4^ cells/well for viability assay), after it adheres to the wall, give Chloroquine (Sigma, USA) of different concentrations (proliferation assay 0-60μM, viability assay 0-480μM). After the effect is over, add 10μl CCK8 reagent (MCE, USA) to incubate for 2 hours, and measure the Optical density (OD) value at 450nm.

### Wound Healing Assay

RKO, HCT-116 and CT26 cells are spread in a 6-well plate at 3×10^5^ cells/well. After they have adhered to a single layer of cells, use the pipette tip to draw a lane in the 6-well plate. Chloroquine was added to a 6-well plate at different concentrations (0-60μM), and photographed with an inverted phase contrast microscope (Nikon, Japan) at 24 hours and 48 hours for recording and analysis.

### Colony Formation Assay

Count the cells, spread 500 cells per well in a 6-well plate, set different concentrations of chloroquine (0-60μM) for treatment, wait for about a week, observe the formation of dot-shaped clones at the bottom of the 6-well plate, fix the cells, stain with crystal violet for 30 minutes and take photos for analysis.

### Transwell Assay

Spread the cells in 5×10^4^ cells in a transwell, and add chloroquine of different concentrations (0-60μM). Place the transwell in a 37°C constant temperature cell incubator for 24-36 hours, take the transwell out of the incubator, fix cells in the transwell with 4% tissue fixative (Solarbio, Beijing, China) for 30 minutes, stain cells in the transwell with crystal violet staining solution for 30 minutes, wipe the upper chamber cells with a cotton swab, observe cells under a microscope, and take pictures.

### Western Blotting

Cells are treated with chloroquine for 12-24 hours, the cell pellet is collected by centrifugation, add lysis buffer (Beyotime Biotechnology, Shanghai, China), and protein is collected. After the animal experiment treatment is over, the subcutaneous tumor is taken out, the tissue is frozen and ground with liquid nitrogen, the lysate is added, and the tissue protein is extracted. Use the BCA protein quantification kit (Beyotime Biotechnology, Shanghai, China) to determine the protein concentration and determine the sample volume. After the electroporation is over, 5% skim milk is blocked at room temperature for 1-2 hours, and the primary antibody is incubated at 4°C overnight. The primary antibodies are as follows: PD-1(1:1000, Abcam, MA), MMP2,Cleaved-caspase-3,PD-L1,CD4,CD8 (1:1000,CST,USA), Bax, Bcl-2, Cytochrome-C(1:1000, Abways, China), and Tubulin (1:3000,Sigma,USA), After incubating for 1 hour with the secondary antibody corresponding to the primary antibody, wash the PVDF membrane (Millpore, USA) and use ECL chemiluminescent solution (Millpore, USA) for chemical imaging. Use Image J software for gray value analysis.

### Flow Cytometry

Flow cytometry to detect cell apoptosis: Spread 2.5×10^6^ cells/well in a 6-well plate, and after they adhere to the wall, treat them with chloroquine for 12-24 hours. Collect the cell pellets when the cell status changes under the microscope, and use the AnnexinV, FITC Apoptosis Detection Kit (Dojindo, Japan) for detection. Flow cytometry to detect immune cells: After the treatment, the mouse spleen cells were separated and red blood cell lysate (Beyotime Biotechnology, Shanghai, China) was added. Resuspend the cells in PBS and count them. Take about 2×10^6^ cells from each experimental group, resuspend them in 200 μl of PBS, and add the corresponding fluorochrome-labeled antibodies (CD3/FITC, CD4/PE and CD8/APC) (all purchased from BioLegend, USA). Incubate for 30 minutes at 4°C in the dark. Flow cytometry analysis of fluorescence intensity.

### Animal Modeling and Treatment

CT26 cells were injected subcutaneously into BALB/c mice at 1×10^6^ cells to establish a mouse colorectal cancer model. Started the first treatment, 7 days after the model was built. The mice were randomly divided into 5 groups, namely PBS group, Scramble (Scr) group, CQ group, siPD-1 group, CQ+siPD-1 group. Starting from day 7, the PBS group was intraperitoneally injected with 100μl of PBS per day, and the CQ group and CQ+siPD-1 group were intraperitoneally injected with 100μL of CQ (50mg/kg) per day. Scr group, siPD-1 group and CQ+siPD-1 group were given intratumoral injection of *Salmonella* carrying empty and siPD-1 (4 x10^5^ CFU in 100 μL of PBS/mouse) on the 8th and 14th day. Record tumor volume and mouse body weight and end of treatment on day 21.

### Immunohistochemistry (IHC) and HE Staining (HE)

After the treatment, remove the mouse subcutaneous tumor, fix it with 4% tissue fixative for 24 hours, embed the tissue and slice it (thickness 4μM). HE staining with hematoxylin and eosin (Beyotime Biotechnology, Shanghai, China). The sections were processed with an immunohistochemistry kit (Zhongshan Jinqiao, Beijing, China). The slides were incubated with antibodies against CD4, CD8, and PD1 at 4°C overnight. The next day, it was incubated with the secondary antibody for 2 hours, then stained with DAB and counterstained with hematoxylin. Two pathologists judge and count the results.

### Terminal-Deoxynucleotidyl Transferase Mediated Nick End Labeling (TUNEL)

According to the instructions of the TUNEL Apoptosis Detection Kit (Green Fluorescence, Abbkine, Wuhan, China), the tissue sections were fluorescently stained deoxyribonucleotide terminal transferase, based on the notch end labeling method. The tissue sections were deparaffinized and dehydrated, incubated with proteinase K for 15 minutes, and incubated with Tunel staining reagent at 37 °C for 1 hour in the dark, and photographed with a confocal microscope (Nikon, Japan).

### Statistics

T-test was used to compare the values between the two groups, and ANOVA was used for three groups and more than three groups. Comparisons were considered to be statistically significant difference when *P <*0.05.

## Results

### Chloroquine Inhibits the Survival and Proliferation of Colon Cancer Cells and Induces Apoptosis

**Figure 1:**
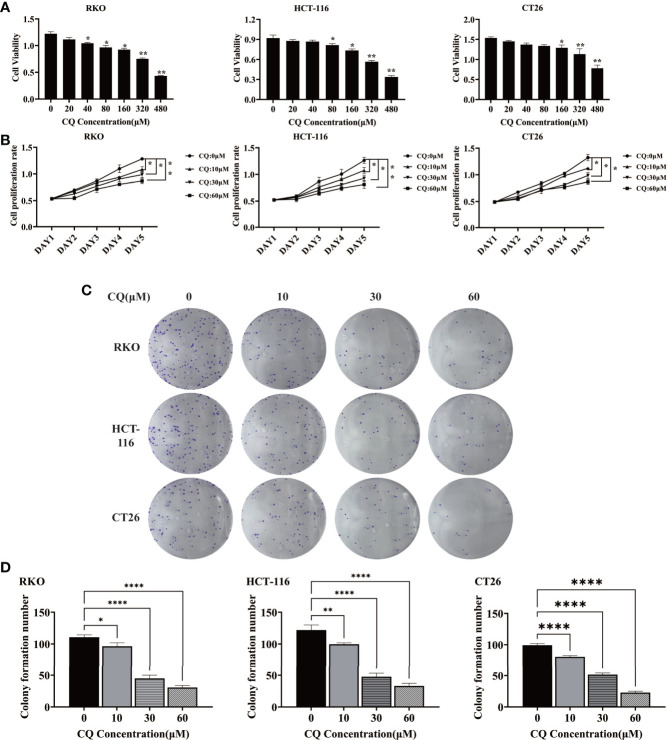
Chloroquine inhibits the survival and proliferation of colorectal cancer cells. **(A)** The cell viability of RKO, HCT-116 and CT26 was detected by CCK8 assay. **(B)** The cell proliferation of RKO, HCT-116 and CT26 was detected by CCK8 assay. **(C, D)** The cell proliferation of RKO, HCT-116 and CT26 was detected by colony-forming assay. (*p < 0.05, **p < 0.01, ****p < 0.0001 *vs* 0μM group).

**Figure 2:**
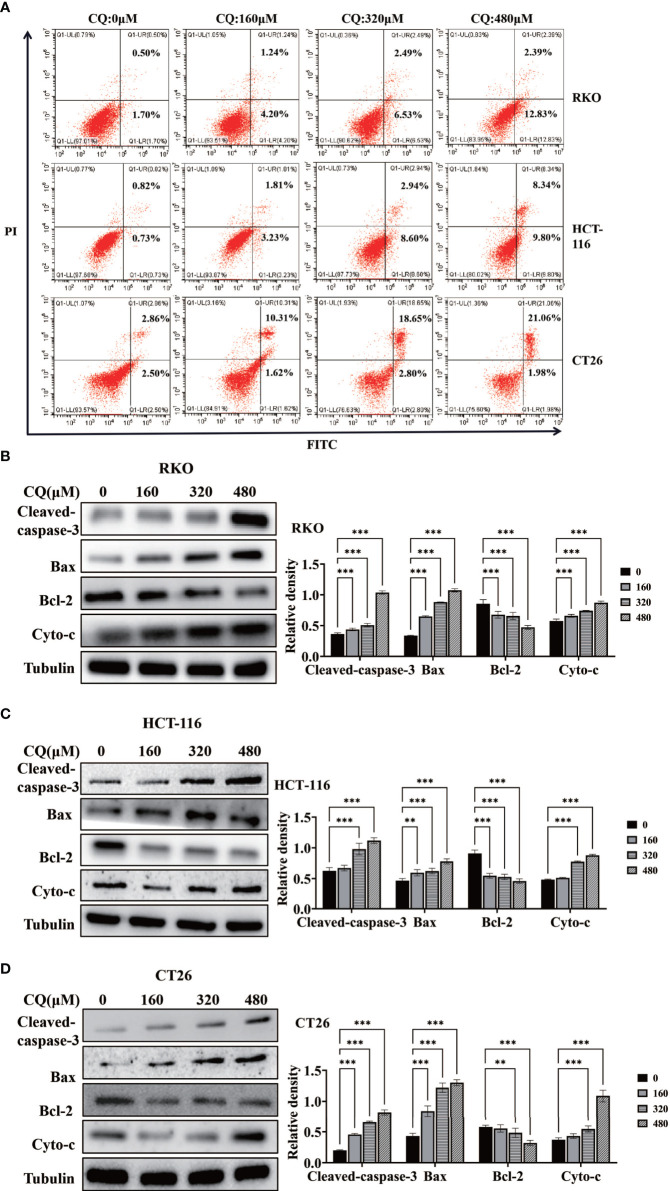
Chloroquine induces apoptosis of colorectal cancer cells and affects the expression of related proteins. **(A)** Flow cytometry detected the apoptotic rate of RKO, HCT-116 and CT26 induced by CQ. **(B–D)** Western blotting analysis for the protein expression of Cleaved caspase 3, Bax, Bcl-2 and Cytochrome c (Cyto-c) after treatment with a chain of concentrations of CQ. (**p < 0.01, ***p < 0.001 *vs* 0μM group).

In order to clarify the effect of chloroquine on the biological behavior of colon cancer cells, we first examined the survival and proliferation of colon cancer cells treated with chloroquine. The results showed that compared with the control group, different concentrations of chloroquine could significantly reduce the survival rate of three kinds of colon cancer cells (**Figure 1A**). Furthermore, CCK8 proliferation test and plate cloning test showed that chloroquine could significantly inhibit the proliferation of colon cancer cells (**Figures 1B–D**). Furthermore, the effect of chloroquine on apoptosis of colon cancer cells was determined by flow cytometry. The results showed that chloroquine could increase the apoptosis rate of three kinds of colon cancer cells (**Figure 2A**). A variety of pathways can induce apoptosis, and mitochondrial apoptosis pathway is the most important one. Our test results showed that chloroquine could significantly affect mitochondrial apoptosis-related molecules, Bax, Bcl-2 and Cytochrome C, and meanwhile induce the expression of apoptosis effector molecule, cleaved caspase-3 (**Figures 2B–D**).

### Chloroquine Inhibits the Migration of Colorectal Cancer Cells

**Figure 3:**
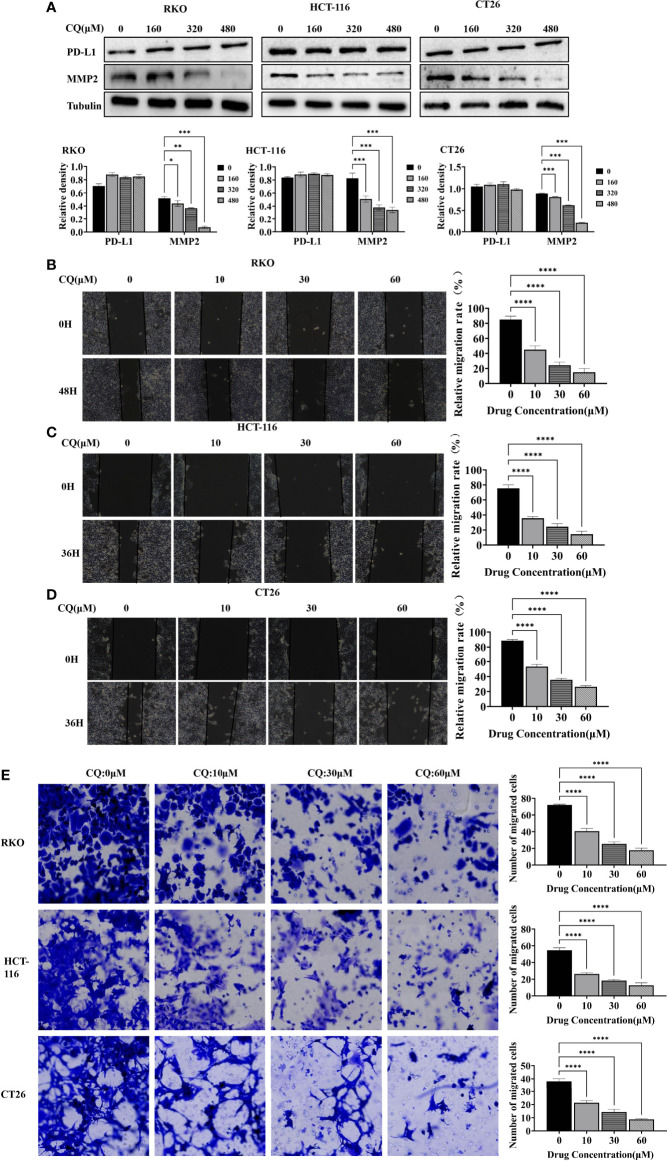
Chloroquine inhibits migration of colorectal cancer cells. **(A)** Western blotting analysis for the protein expression of PD-L1, MMP2 after treatment with a chain of concentrations of CQ. **(B–D)** Wound healing assay detects the effect of different concentrations of CQ on the migration ability of colorectal cancer cells RKO, HCT-116 and CT26. **(E)** Transwell assay detects the effects of different concentrations of CQ on the migration ability of colorectal cancer cells RKO, HCT-116 and CT26. (*p < 0.05, **p < 0.01, ***p < 0.001, ****p < 0.0001 *vs* 0μM group).

We further used the wound healing assay and the transwell assay to detect the effect of chloroquine on the migration ability of colon cancer cells RKO, HCT-116 and CT26. The results showed that as the concentration increased, the migration ability of colon cancer cells decreased (**Figure 3**). Meanwhile, western blotting experiment was used to detect the change of the migration phase protein (MMP2), and the results showed that high concentration of chloroquine significantly reduced the expression of MMP2 (**Figure 3A**). The results above indicate that chloroquine can inhibit the migration ability of colorectal cancer cells. However, we found that chloroquine did not affect the expression of PD-L1 in colon cancer cells (**Figure 3A**).

### Chloroquine Up-Regulates the Expression of PD-1 in Tumor Tissues, and the Combination of Chloroquine and PD-1 siRNA Can Further Inhibit Tumor Growth

**Figure 4:**
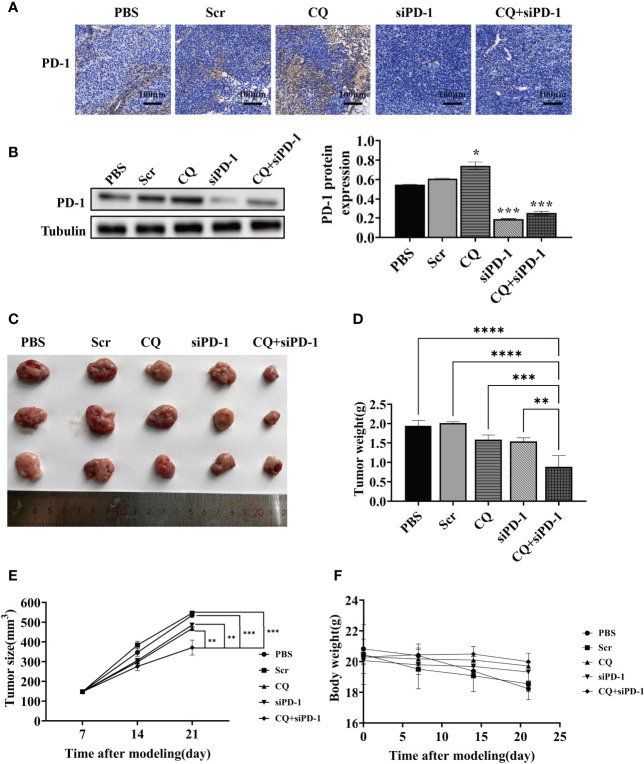
Effect of various treatments on tumor growth in *vivo*. BALB/c mice were subcutaneously inoculated with 1×10^6^ CT‐26 cells and treated with PBS, attenuated *Salmonella* carrying the scrambled siRNA plasmid, CQ, attenuated *Salmonella* carrying PD‐1 siRNA, or CQ plus attenuated Salmonella carrying PD‐1 siRNA. **(A)** IHC detects the expression of PD-1 in tumor tissues. **(B)** The protein levels of PD-1 in tumor tissue after the indicated treatments were evaluated by Western blotting analysis. **(C)** Images of the representative tumors in each group on posttreatment day 14. **(D)** Tumor weight analysis. **(E)** Tumor size analysis. **(F)** Body weight analysis. (*p < 0.05, **p < 0.01, ***p < 0.001, ****p < 0.0001 *vs* CQ+siPD-1 group).

Based on the fact that chloroquine did not affect the expression of PD-L1 in colon cancer cells, we determined whether chloroquine could affect the expression of PD-1 in tumor tissues. IHC results showed that chloroquine could significantly increase the expression of PD-1 in tumor tissues *in vivo*, mainly on the surface of lymphocytes. Western blotting results also showed that chloroquine up-regulated the expression ofPD-1 in tumor tissues (**Figures 4A, B**). Meanwhile, the results showed that the PD-1 siRNA delivered with attenuated *Salmonella* could significantly reduce the expression of PD-1 in tumor tissues. Tumor tissue weight and size test results showed that chloroquine could inhibit the growth of tumor to a certain extent, but chloroquine combined with PD-1 siRNA had the most obvious inhibition (**Figures 4C–E**). The weight of the mice was measured, and the results showed western blotting that there was no significant change, indicating that the combination medication did not have significant side effects (**Figure 4F**).The results suggested that chloroquine and PD-1 blockade synergistically elicit anti-tumor effects.

### The Combination Treatment of Chloroquine and PD-1 siRNA Can Significantly Induce Apoptosis and Inhibit Migration in Colon Cancer Xenografts

**Figure 5:**
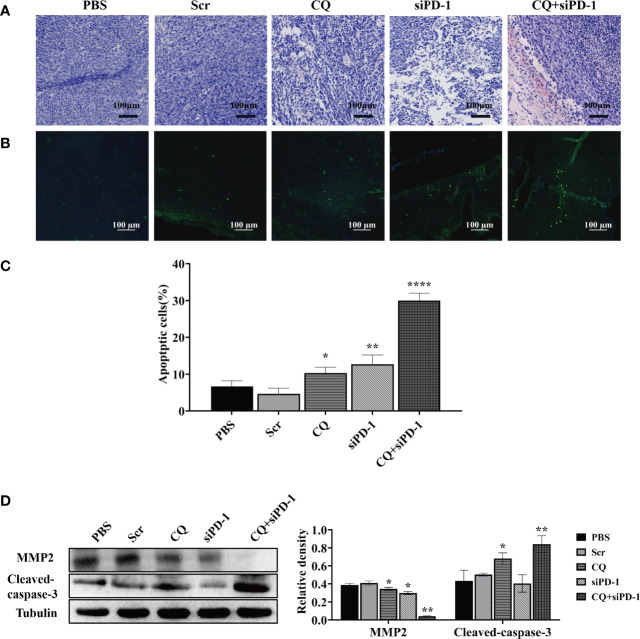
*In vivo* experiments have detected that chloroquine combined with siPD-1 in the treatment of colorectal cancer can significantly inhibit the migration of colorectal cancer and promote cell apoptosis. Tumor tissues were collected on day 14 after the first treatment and were subjected to HE, TUNEL staining, and Western blotting analysis. **(A)** Tissue HE staining. **(B, C)** TUNEL staining and quantitative analysis of tissues. **(D)** The expression of proteins associated with apoptosis and migration.(*p < 0.05, **p < 0.01, ****p < 0.0001 *vs* PBS or Scr group).

To explore the effect of chloroquine on apoptosis and metastasis of colon cancer cells *in vivo*, the hematoxylin-eosin staining (HE staining), transferase-mediated deoxyuridine triphosphate-biotin nick end labeling (TUNEL), and western blotting were used to detect apoptosis and protein expression. As shown in HE staining, tissue disorder or necrosis was observed in the CQ, siPD-1 and CQ+siPD-1 groups, with the most severe damage in the combination treatment group (**Figure 5A**). TUNEL results showed that there were more apoptotic cells in tumor tissues in the combination treatment group (**Figures 5B, C**). Western blotting analysis showed that compared with other groups, the combination treatment group could significantly inhibit the expression of MMP2 and induce the expression of apoptotic protein, cleaved caspase-3 (**Figure 5D**). The results above indicate that CQ combined with PD-1 siRNA can significantly promote the apoptosis of colorectal cancer cells and inhibit migration.

### The Combination Treatment of Chloroquine and PD-1 siRNA Significantly Enhance the Infiltration of Effector T Cells Into Tumor Tissues

**Figure 6:**
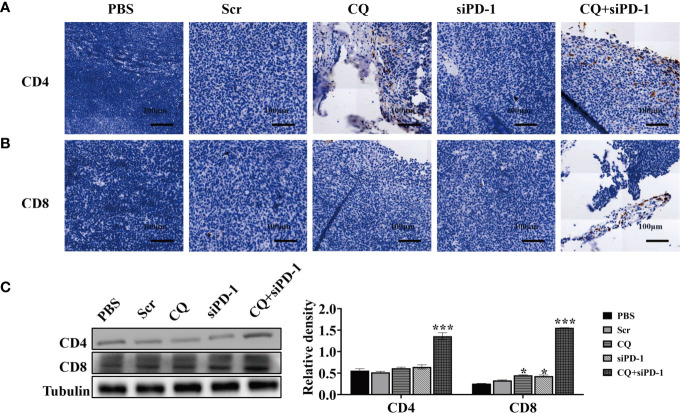
Chloroquine combined with PD-1 siRNA can promote the infiltration of T cells into tumor tissues and inhibit the expression of PD-1. **(A, B)** IHC detects the expression of CD4^+^ and CD8^+^ in tumor tissues. **(C)** The protein levels of CD4 and CD8 in tumor tissue after the indicated treatments were evaluated by Western blotting analysis. (*p < 0.05, ***p < 0.001 *vs* PBS or Scr group).

Furthermore, we used immunohistochemistry (IHC) to detect the expression of CD4, CD8 and PD-1 in colorectal cancer tumor tissues. The results showed that compared with other groups, the expression of CD4 and CD8 in the combination treatment group was higher (**Figures 6A, B**). Western blotting analysis showed that treatment of CQ combined with PD-1 siRNA could increase the expression of CD4 and CD8 (**Figure 6C**). In summary, these results indicate that the combination treatment of CQ and PD-1 siRNA can enhance the infiltration of CD4^+^ and CD8^+^ T lymphocytes in colon cancer tissues.

### Combination Treatment With Chloroquine and PD‐1 siRNA Elicited Systemic and Synergetic Antitumor Immunity

**Figure 7:**
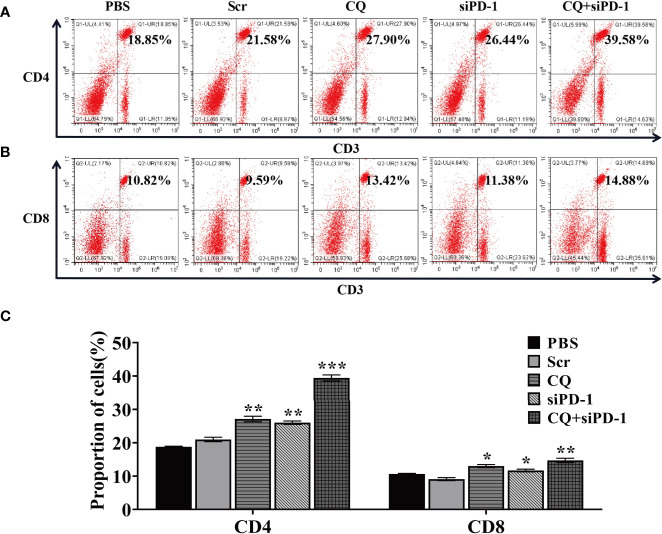
The combination treatment of chloroquine and PD-1 siRNA can regulate the body’s anti-tumor immunity. **(A, B)**, Flow cytometry detects the proportion of CD4^+^ and CD8^+^ T cells in cells extracted from tumor tissue. **(C)** Data analysis of the ratio of cells positive for each surface marker. (*p < 0.05, **p < 0.01, ***p < 0.001 *vs* PBS or Scr group).

In order to determine whether the anti-tumor effect of chloroquine and PD-1 siRNA combined therapy is due to the activation of systemic tumor-specific cellular immunity, we isolated T lymphocytes from mouse spleen cells of all groups, respectively. The results showed that the combination therapy significantly prolonged the percentage of CD4^+^ and CD8^+^ T cells (**Figure 7**). These results indicate that the increase in T cell infiltration may be due to systemic and synergetic anti-tumor immunity exerted by chloroquine and PD‐1 siRNA.

## Discussion

With the emergence of chemotherapeutic drug resistance and the in-depth research on the mechanism of drug action, many traditional non-chemotherapeutic drugs are used in the treatment of cancer, such as aspirin and metformin, which are called old drug in new use (30–33). Chloroquine, with the molecular formula, C18H26ClN3, is an anti-malarial and anti-inflammatory agent widely used in the treatment of malaria and rheumatoid arthritis (34, 35). In addition, chloroquine is often known as an inhibitor of the end of autophagy, which inhibits the degradation of substances in autophagic vesicles by increasing the potential of hydrogen (pH) of the lysosome, thereby causing cell damage (36). Studies have shown that chloroquine can induce and inhibit tumors, but these studies were mainly focused on proliferation and apoptosis, and the effect of chloroquine on cell migration and invasion is not very clear (37, 38). Our results show that chloroquine can inhibit the survival, proliferation and induce apoptosis of colon cancer cells, and at the same time can significantly inhibit the invasion and migration of colon cancer cells.

A large number of studies have shown that chemotherapy or radiotherapy alone has little benefit to cancer patients after surgery (39–41). Combination therapies have been widely researched and clinically recognized (42, 43). Among them, the most extensive research in recent years focused on the combination immunotherapy (44, 45). There are many kinds of immunotherapies, including vaccines, antibodies, cytokines, cell therapies and immunosuppressive agents. Among them, the most widely studied and the most recognized is the treatment based on immune checkpoints (46). At present, the most widely applied is the PD-1/PD-L1 monoclonal antibody, and it shows a significantly better effect than chemotherapy alone (47, 48). This brings new hope to cancer patients. Although there are many studies focused on the anti-tumor effect of chloroquine, there are few studies centered on the relationship between chloroquine and tumor immune response. We first tested the effect of chloroquine on the expression of PD-L1 in tumor cells and found that chloroquine could not significantly affect the expression of PD-L1 in tumor cells. In order to explore this problem, we established a subcutaneous transplanted-tumor model of colon cancer in mice, and further observed its effect on the expression of immune cells PD-1 in tumor tissues. The results showed that the administration of chloroquine could significantly increase the expression of PD-1 in tumor tissues. At the same time, we found that the number of CD4^+^ and CD8^+^ T cells in tumor tissues increased significantly. This phenomenon has a dual effect. On one hand, it increases the infiltration of lymphocytes in tumor tissues, which is beneficial to the killing effect of immune cells; on the other hand, the disadvantage is that the increase of PD-1 can inhibit the killing effect of immune cells. Based on this, we used our pre-designed PD-1 siRNA and chloroquine to act on colon cancer tissues. The results found that compared with chloroquine alone, the tumor suppression in the chloroquine combined with PD-1 siRNA group was very obvious, showing a combination effect. On the basis of inhibiting tumor growth, we detected the expression of apoptosis and metastasis-related proteins in tumor tissues. The expression level of apoptosis in the combination treatment group was significantly increased, and at the same time the expression level of the metastasis-related protein,MMP2, was significantly inhibited. To explore the change in the number of immune cells in tumor tissues, we further detected the number of CD4^+^ and CD8^+^ T cells in the spleens of mice, and found that the combination treatment group could also significantly increase the number of immune cells in the spleens. These results show that the combination use of chloroquine and PD-1 siRNA can effectively activate the immune response, thereby inhibiting tumors.

Compared with monoclonal antibodies and small molecule inhibitors, the use of siRNA to treat tumors requires an effective delivery system. In addition to viral vectors, recent studies have shown that a variety of bacteria can be used as carriers, among which attenuated *Salmonella* is the most widely studied and applied (49, 50). As a vehicle, attenuated *Salmonella* can carry small hairpin RNA (shRNA ) to a variety of tumor sites, including lung cancer, breast cancer, gastric cancer and prostate cancer. The advantages of attenuated *Salmonella* as a carrier are: on one hand, it can target the low-oxygen environment of solid tumors; on the other hand, it can stimulate an effective immune response. In this study, we used phoP/phoQ-deleted *Salmonella Typhimurium* to effectively carry PD-1 siRNA to tumor tissues, and at the same time, activate a robust immune response, exhibiting a good application prospect.

**Figure 8:**
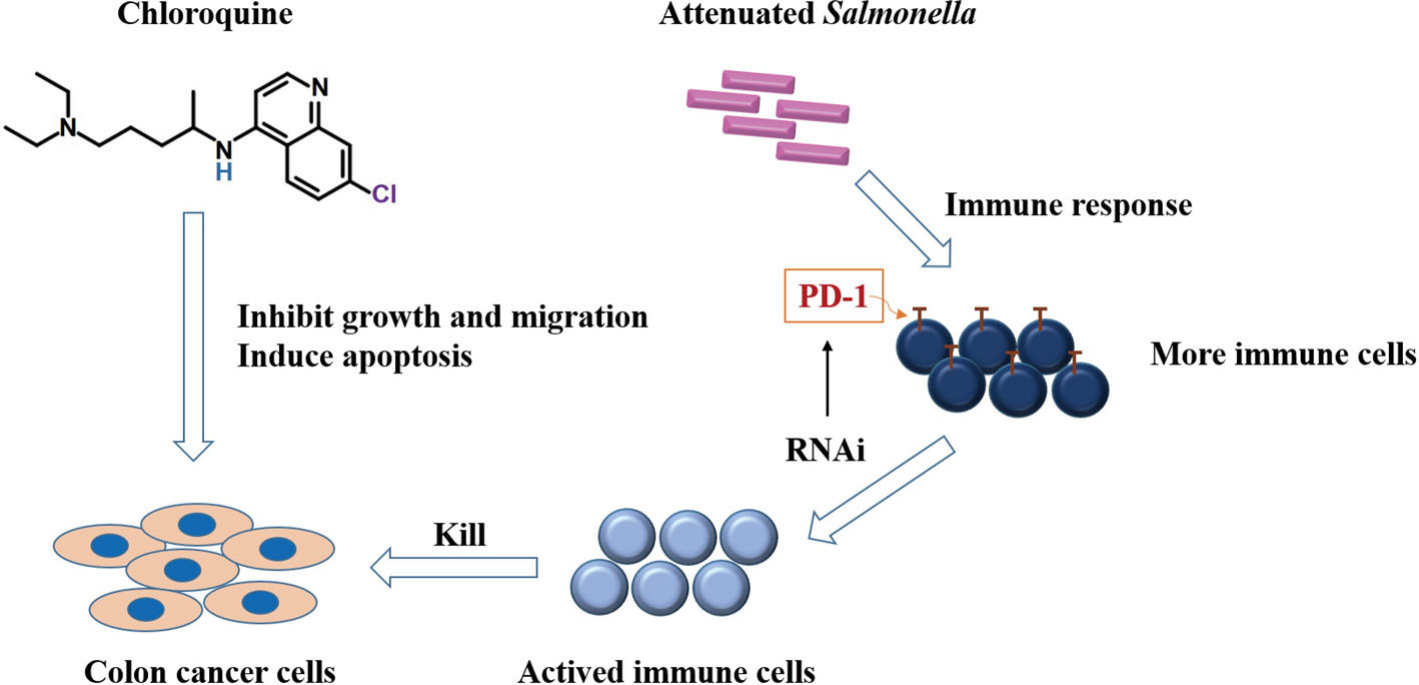
Schematic diagram demonstrating the potential mechanism of chloroquine and PD-1 siRNA in combination.

This study clarified that chloroquine could significantly inhibit colon cancer cell proliferation and induce apoptosis while inhibit its migration and invasion capabilities. Combining chloroquine and PD-1 siRNA delivered with attenuated *Salmonella* could effectively inhibit the growth of tumors and had a good synergistic effect. The mechanism was due to the killing effect of chloroquine on tumor cells, the effective immune response and the increases in the number of immune cells. At the same time, siRNA further suppressed the immunosuppressive molecule, PD-1, on the surface of immune cells (**Figure 8**). The research provided a basis and new direction for the clinical application of chloroquine and immunosuppressive agents combined to treat tumors more validly in the future.

## Data Availability Statement

The original contributions presented in the study are included in the article/supplementary material. Further inquiries can be directed to the corresponding authors.

## Ethics Statement

The animal study was reviewed and approved by Ethics Committee of Xinxiang Medical University.

## Author Contributions

JZ, TZ, JG, and MW designed the experiments and wrote the manuscript. SL, JG, HJ, YL, YD, FS, ZL, and SM performed the experiments. SL, JG, and HJ contributed equally to this work. All authors contributed to the article and approved the submitted version.

## Funding

This work was supported by the National Natural Science Foundation of China (No.81702891 and U1804173), Zhongyuan Qianren Jihua of Henan Province (No. ZYQR201810153), Joint construction project of Henan Medical Science and technology research plan (No. LHGJ20190452), Natural Science Foundation of Henan Province (No. 202300410326), Science and Technology Program foundation of Henan Province, China (No.202102310091 and 172102310651), Henan University Science and Technology Innovation Team Support Program (20IRTSTHN030), Outstanding Youth Project of Henan Natural Science Foundation (212300410013), the Young Backbone Teacher Training Projects of Universities in Henan province (2020GGJS149), the Key Projects of Scientific Research for Higher Education of Henan Province (21A310012), Innovative talents of science and technology in Colleges and universities of Henan Province (20HASTIT043).

## Conflict of Interest

The authors declare that the research was conducted in the absence of any commercial or financial relationships that could be construed as a potential conflict of interest.
